# Development of New Composite Materials by Modifying the Surface of Porous Hydroxyapatite Using Cucurbit[n]urils

**DOI:** 10.3390/ma17092041

**Published:** 2024-04-26

**Authors:** Tolkynay Burkhanbayeva, Arthur Ukhov, Dmitry Fedorishin, Alexander Gubankov, Irina Kurzina, Abdigali Bakibaev, Rakhmetulla Yerkassov, Togzhan Mashan, Faiziya Suyundikova, Nurgul Nurmukhanbetova, Aina Khamitova

**Affiliations:** 1 Department of Chemistry, L.N. Gumilyov Eurasian National University, Astana 010008, Kazakhstan; erkass@mail.ru (R.Y.); togzhan-mashan@mail.ru (T.M.); sfaiziya@mail.ru (F.S.); 2 Faculty of Chemistry, National Research Tomsk State University, Arkady Ivanov St. 49, 634028 Tomsk, Russia; artyryxov1@gmail.com (A.U.); strix187@yandex.ru (D.F.); 4.4.gub4nk0v@gmail.com (A.G.); kurzina99@mail.ru (I.K.); bakibaev@mail.ru (A.B.); 3 Department of Chemistry and Biotechnology, Ualikhanov University, Abaya St. 76, Kokshetau 020000, Kazakhstan; nn_nurgul@mail.ru (N.N.); aina-hamitova@mail.ru (A.K.)

**Keywords:** biocompatible materials, hydroxyapatite, cucurbit[n]urils, cytotoxicity, hemolytic activity, anti-inflammatory effect

## Abstract

This study represents an advancement in the field of composite material engineering, focusing on the synthesis of composite materials derived from porous hydroxyapatite via surface modification employing cucurbit[n]urils, which are highly promising macrocyclic compounds. The surface modification procedure entailed the application of cucurbit[n]urils in an aqueous medium onto the hydroxyapatite surface. A comprehensive characterization of the resulting materials was undertaken, employing analytical techniques including infrared (IR) spectroscopy and scanning electron microscopy (SEM). Subsequently, the materials were subjected to rigorous evaluation for their hemolytic effect, anti-inflammatory properties, and cytotoxicity. Remarkably, the findings revealed a notable absence of typical hemolytic effects in materials incorporating surface-bound cucurbit[n]urils. This observation underscores the potential of these modified materials as biocompatible alternatives. Notably, this discovery presents a promising avenue for the fabrication of resilient and efficient biocomposites, offering a viable alternative to conventional approaches. Furthermore, these findings hint at the prospect of employing supramolecular strategies involving encapsulated cucurbit[n]urils in analogous processes. This suggests a novel direction for further research, potentially unlocking new frontiers in material engineering through the exploitation of supramolecular interactions.

## 1. Introduction

In the contemporary era of personalized medicine, there exists a pressing demand for the development of novel materials tailored for implants, each mandated to possess specific characteristics. To ensure their efficacy in medical applications, such materials necessitate an optimal blend of diverse properties [1]. As the demand for precise and tailored treatments increases, ongoing research focuses on enhancing traditional biomaterials for established applications while also seeking new bioactive materials suitable for diverse advanced applications, such as regenerative medicine and addressing various health issues, including theranostic biomaterials (offering diagnostic, monitoring, and combined therapeutic capabilities). In contemporary medical practice, biomaterials play a significant role, serving to stimulate healing and facilitate the restoration of initial biological or functional activity. These biomaterials may be derived from natural sources or synthetically produced [2].

The fundamental attributes of these materials encompass their composition, shape, structure, and mechanical properties. Biocompatibility emerges as a critical facet, denoting the capacity to interface with surrounding tissues without provoking adverse reactions or inflammation. Moreover, it is advantageous for such materials to promote the proliferation of blood vessels or bones in their vicinity, thereby facilitating the healing and regeneration of impaired tissues. Whether employed in implants or wound treatment, these properties delineate the material’s capability to interface with adjacent tissues. Bioactive materials hold the potential to serve as a scaffold for tissue regeneration, hastening the repair of damage [1].

Conversely, materials in direct contact with the body’s internal environment ought to exhibit minimal toxicity towards cells and tissues. Nevertheless, numerous substances boasting potent antibacterial attributes often carry significant toxicity, thereby compromising their biocompatibility. This underscores the intricate nature of the interaction between biomaterials and host tissues, representing a paramount challenge in biomaterials research [3]. Biocompatibility refers to the immune rejection or inflammatory response of the surrounding tissue systems triggered by the presence of a foreign body within the body [3].

In adherence to the rigorous clinical standards governing medical device deployment, a meticulous surface modification protocol stands as an imperative prerequisite prior to implantation within the human body, aiming to bolster biocompatibility. Consequently, the quest for materials exhibiting dual characteristics of biocompatibility and specific biological functionality presents a formidable scientific endeavor [4].

Moreover, modern clinical therapeutic strategies hinge upon the administration of drugs via oral ingestion or intravenous infusion, facilitating rapid elevation of drug concentration in the bloodstream following dosage administration. However, this concentration swiftly diminishes below the therapeutic threshold. Consequently, there is a risk of the drug level spiking to toxic levels post-administration, followed by a drop below the therapeutic range, rendering the therapy ineffective [4].

The advent of drug-releasing implants presents a compelling departure from traditional routes of drug delivery, such as oral ingestion and intravenous infusion, offering an array of promising applications across diverse clinical treatments. Key materials currently under exploration for the fabrication of such implants include titanium nanotubes, porous silicon, polymers, hydrogels, and microtechnologies [5].

Drug-releasing implants hold immense potential for enabling sustained, remotely controlled, programmable, and localized drug release at specific sites within the body. This capability not only enhances therapeutic effectiveness but also minimizes adverse effects experienced by patients. These attributes significantly surpass the capabilities of conventional systemic drug administration methods, marking a significant stride in the field of therapeutic interventions [5].

Bioactive materials hold a unique position among implants due to their capacity to promote the growth of new tissue in their vicinity. This capability facilitates enhanced healing and recovery processes, making them particularly crucial for medical applications pertaining to wound and injury treatment [6].

All these attributes of implant materials play a vital role in their application in medical practice. Ongoing research and development in this domain is continually advancing, aiming to identify the most suitable materials that fulfill all prerequisites and deliver optimal outcomes for patients [6].

There exists a necessity to devise materials that engage with the body’s internal environment while imposing minimal toxicity on cells and tissues. Nonetheless, numerous substances endowed with antibacterial properties can exert significant toxicity on living tissues. Consequently, a dilemma frequently arises: heightened antibacterial efficacy coincides with diminished biocompatibility. This complicates the challenge of crafting materials possessing both antibacterial attributes and robust biocompatibility [6].

One potentially promising material is modified hydroxyapatite (**HA**) augmented with biologically active compounds. Hydroxyapatite constitutes a pivotal component of bones (comprising roughly 50% of total weight) and teeth (constituting 96% of enamel) and can exist in both synthetic and natural forms.

In the medical realm, synthetic hydroxyapatite (**HA**) serves multifaceted roles, including as a filler for the restoration of bone defects and as a coating on implants to facilitate the regeneration of new bone tissue [7]. Additionally, **HA** finds application as a constituent in biocomposites and as coatings for surgical implants. Notably, these materials exhibit inertness towards living tissues and demonstrate low toxicity levels [7].

Furthermore, the utilization of synthetic hydroxyapatites is associated with a minimal risk of eliciting allergic reactions, inflammation, or mutagenic effects. Modified forms of hydroxyapatites have been demonstrated to expedite the process of reparative osteogenesis at the implantation site while concurrently augmenting osteoblast proliferation. These attributes collectively underscore the pivotal role of synthetic hydroxyapatites in advancing regenerative medicine and orthopedic surgery [8].

To date, techniques for the surface modification of porous materials through saturation with biologically active compounds, such as macrocyclic compounds, are gaining prominence. These methods afford the ability to regulate the release of antibiotics, drugs, biologically active substances, and cells [9].

Macrocyclic compounds are frequently favored over alternative drug delivery systems, like dendrimers, liposomes, micelles, carbon nanotubes, hydrogels, and polymers. This preference stems from several advantages that they offer [10,11,12]. Macrocyclic compounds typically exhibit enhanced stability and enable a controlled rate of drug release [10,11,12]. The development of efficient drug and biologically active substance delivery systems represents a significant stride in advancing novel approaches in medicine and various other domains of science and technology [10,11,12].

Macrocyclic systems derived from glycoluril and its derivatives, such as cucurbit[n]urils and bambusurils, serve as ideal starting reagents for surface modification of porous materials. In contrast to cucurbit[n]urils (**CB[n]**), bambus[6]uril (**BU[6]**) exhibits a relatively weaker affinity for hydrogen bonding with anions situated within its hydrophobic cavity [13]. The positively charged electrostatic domain of **BU[6]** facilitates the attraction of anions, while the portal carbonyl oxygen atoms generate a negative region capable of interacting with positively charged particles. Our investigations focused on evaluating the hemocompatibility and the capacity for human plasma protein adsorption of materials incorporating **BU[6]** and hydroxyapatite [14].

Modification of **BU[6]** samples resulted in a reduction in protein adsorption, thereby enhancing their hemocompatibility. This phenomenon is likely attributed to alterations in the surface properties of the material induced during **BU[6]** modification, including changes in surface tension, free surface energy, roughness, and hydrophilicity. Deposition of **BU[6]** onto the surface of porous materials is speculated to induce modifications in surface charge, mimicking characteristics akin to blood [14]. Consequently, the diminished plasma protein adsorption mitigates thrombogenicity, thereby augmenting hemocompatibility [14].

In contrast to **BU[6]**, **CB[n]** showcases a distinct capacity for forming host–guest complexes with cationic molecules and boasts a broader spectrum of molecular cavity sizes [15]. **CB[n]** displays an inherent ability to selectively accommodate diverse organic guest molecules through a repertoire of interaction mechanisms including hydrophobic interactions, hydrogen bonding, Van der Waals forces, π-π stacking, and ion–dipole effects, culminating in the formation of inclusion complexes [16,17].

The utilization of **CB[n]**’s hydrophobic cavity in assembling biologically active functional molecules has garnered considerable attention among researchers. This interest extends beyond its role as a targeted drug delivery system to encompass applications in disease diagnosis and various other domains [18,19]. Unlike many other macrocyclic hosts, **CB[n]** exhibits an exceptionally rigid structure, rendering the determination of cavity parameters particularly elucidative. For instance, all **CB[n]** variants (n = 5–8 and 10) share a uniform height (d = 9.1 Å) while manifesting significant discrepancies in cavity width. **CB[5]** has an inner diameter of 4.4 Å, while **CB[8]** has twice that size at 8.8 Å [20,21]. This characteristic enables the accommodation of drug molecules of varying sizes within the **CB[n]** cavity, thereby expanding its potential applications across various fields. Presently, antitumor, antibacterial, anticholinergic drugs, antioxidants, neurotransmitters, cholinesterase reactivators, and other compounds are being encapsulated within the **CB[n]** cavity [22]. Leveraging these properties enables researchers to impart various necessary characteristics into materials. Currently, techniques for surface modification of porous materials through impregnation with biologically active compounds, including macromolecular entities, are experiencing widespread adoption for various applications [22]. These methodologies offer meticulous control over the release kinetics of antibiotics, pharmaceutical agents, biologically active substances, and cells, thus presenting versatile avenues for tailored therapeutic interventions and biomedical applications [9].

In summary, the utilization of nitrogen-containing macrocyclic compounds for the surface modification of porous materials serves two primary objectives: enhancing biocompatibility and enabling the development of medical biomaterials with controlled drug release capabilities. Consequently, **CB[n]** emerges as unique and intriguing molecules for the fabrication of biocompatible materials with multifaceted applications across various fields. Continued research in this domain holds the potential to further broaden the horizons of their utilization and uncover new breakthroughs.

This study marks the inaugural deposition of a series of cucurbit[n]urils (**CB[6]**, **CB[7]**, and **CB[8]**) onto the surface of hydroxyapatite (**HA**). This innovative approach serves as a foundation for saturating the cavities of cucurbit[n]urils with therapeutic agents, thereby imparting therapeutic functionality to hydroxyapatite and facilitating osteogenesis. Accordingly, the primary aim of this investigation is to assess the influence of cucurbit[n]uril precipitation on the structural integrity of hydroxyapatite (**HA**) and to evaluate the in vitro biocompatibility of the resultant biocomposite materials.

## 2. Materials and Methods

All chemicals were from Merck/Sigma-Aldrich (Darmstadt, Germany).

### 2.1. Instruments for Interpreting the Results

#### 2.1.1. Fourier Transform Infrared (FTIR) Spectroscopy

The identification and structural investigation of the obtained samples were conducted using Fourier transform infrared (FTIR) spectroscopy on a Nicolet 6700 IR spectrometer manufactured by Thermo Fisher Scientific (Waltham, MA, USA). The samples underwent examination utilizing the attenuated total internal reflection (ATR) method within the spectral range of 400 to 4000 cm^−1^, with a resolution of 4 cm^−1^. The reflection spectra obtained were converted into absorption spectra utilizing the Kubelka–Munk transform.

#### 2.1.2. Nuclear Magnetic Resonance (NMR) Spectroscopy

The NMR spectra of the obtained **CB[6]**, **CB[7]**, and **CB[8]** were acquired using a Bruker Avance 400 III HD NMR spectrometer (Billerica, MA, USA). The measurements were performed in DMSO-d6 solution at a temperature of 25 °C. The operating frequency for hydrogen nuclei was 400 MHz, while for carbon nuclei, it was 100 MHz. These NMR spectra provide valuable information about the molecular structure and chemical environment of the cucurbit[n]uril compounds, aiding in their characterization and analysis.

#### 2.1.3. Scanning Electron Microscopy (SEM) Samples **CB[n] + HA**

The QUANTA 200 3D (Hillsboro, OR, USA) electronic and focused beam system is equipped with versatile capabilities for electron microscopy and imaging. It features a wide range of accelerating voltages from 200 to 30,000 V continuously, allowing for flexible adjustments to suit various imaging requirements. In ESEM mode, it achieves a resolution of 3.5 nm at 30 kV, enabling high-quality imaging even under environmental conditions. Additionally, in low vacuum mode, it can achieve a resolution of less than 15 nm at 3 kV, facilitating detailed imaging with enhanced contrast and reduced beam damage. This system is well-suited for a range of applications in materials science, nanotechnology, and biological research.

#### 2.1.4. X-ray Diffraction (XRD)

The crystalline powders (**CB[6]**, **CB[7]**, and **CB[8]**) were analyzed using X-ray phase analysis. The examination was conducted utilizing an XRD-7000 X-ray diffractometer manufactured by Shimadzu, Kyoto, Japan. The instrument utilizes a copper (Cu) anode and a radiation wavelength of Ka(Cu) = 1.5406 Å. The measuring range extended from 5 to 50° in 2θ, with a measuring speed of 30°/min. Identification of the analyzed samples was accomplished by comparing the spectrum with the diffractogram of reference substances using diffraction data from the Cambridge Crystallographic Data Centre database. This technique allows for the determination of the crystalline structure and phase composition of the samples.

### 2.2. Getting Scaffolds from **HA**

Hydroxyapatite (**HA**) synthesis was carried out by the liquid–phase method using microwave radiation at pH~11 according to the following scheme [23]:

10Ca(NO_3_)_2_ + 6(NH_4_)_2_HPO_4_ + 8NH_4_OH → Ca_10_(PO_4_)_6_(OH)_2_ + 20NH_4_NO_3_ + 6H_2_O

To prepare the initial solutions for hydroxyapatite synthesis, the following chemicals were used:

1. Calcium nitrate tetrahydrate (Ca(NO_3_)_2_·4H_2_O): This compound serves as the calcium precursor in the synthesis process. It is typically dissolved in distilled water to obtain a calcium-containing solution.
2. Ammonium phosphate ((NH_4_)_2_HPO_4_): Ammonium phosphate acts as the phosphate precursor in the synthesis reaction. It is dissolved in distilled water to prepare a phosphate-containing solution.
3. Twenty-five percent aqueous ammonia solution: This solution is used to adjust the pH of the reaction mixture to around 11, creating suitable conditions for hydroxyapatite formation.
4. Distilled water: Distilled water is used as a solvent to dissolve the calcium nitrate tetrahydrate and ammonium phosphate precursors, as well as for dilution purposes and to ensure the purity of the reaction components.

**Figure 1:**
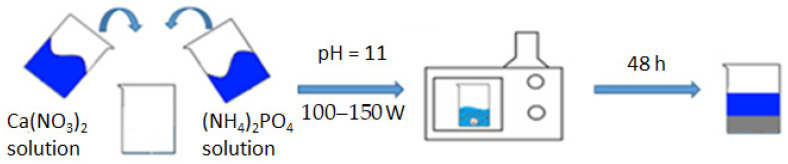
Synthesis scheme of **HA**.

By accurately preparing and combining these chemical solutions in the appropriate proportions, researchers can initiate the hydroxyapatite synthesis reaction under microwave irradiation to obtain the desired **HA** product (Figure 1).

A suspension of m = 47.20 g of Ca(NO_3_) was dissolved in a beaker (300 mL)^2^ × 4H_2_O with distilled water (V = 200 mL), obtaining a solution with a concentration of 0.5 mol/L; we similarly dissolved m = 15.84 g (NH_4_)_2_HPO_4_ in 200 mL of distilled water, obtaining a solution with a concentration of 0.3 mol/L. The prepared solutions were mixed in one 500 mL glass and brought to pH = 11, adding 25% aqueous ammonia solution with constant stirring. The glass with the reaction mixture was covered with a film and placed in a microwave oven; an operating power of 100–150 watts was set, and heating was turned on. The microwave exposure was carried out until the reaction mixture boiled. The contents of the reaction vessel were periodically stirred every 15 min of microwave synthesis in order to avoid local overheating. The total heating time in the microwave was 45 min. After that, the glass with its contents was removed and left at room temperature to form the hydroxyapatite phase for 48 h. The precipitated **HA** was filtered and dried in a drying cabinet at 110 °C to a constant weight (20 h), after which it was milled to a homogeneous state. From the resulting **HA** powder, carriers were formed in tablet form with a width of 2 cm and a thickness of about 1 mm, and then calcined at 600 °C to a constant mass and sintering of the material. For this standard technique, the physico-chemical parameters of the resulting compound do not practically differ from the literature data, namely in terms of particle sizes 0.5–1 nm, specific area, m^2^/g^−1^ = 106.0, total pore volume, cm^3^/g^−1^ = 0.50, and their average size, which is 20 nm [6].

**Figure 2:**
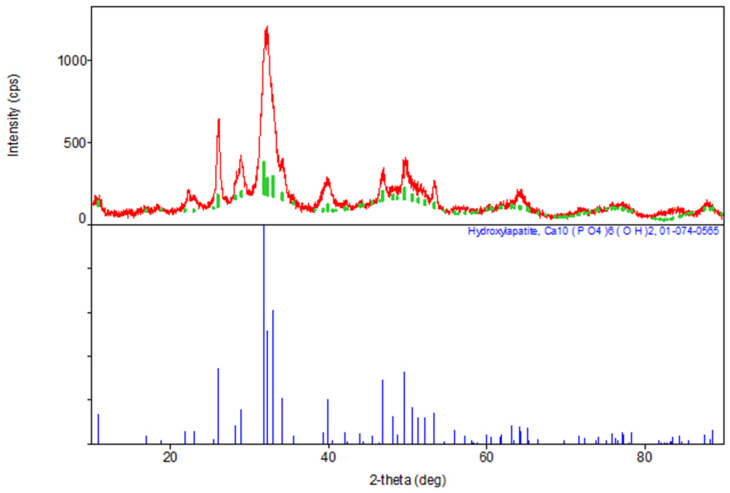
Diffractogram of synthesized stoichiometric **HA**, red color—obtained substance, green—standard.

The results of the XRD indicate the formation of a hexagonal syngony, gr. P63/m, during the synthesis of stoichiometric **HA**, the gross formula of which can be described by the formula Ca_10_(PO_4_)_6_(OH)_2_ (Table 1). The parameters of the synthesized **HA** cell are close in values to the tabular data (JCPDS data, No. 9-432 [6]) (Figure 2).

### 2.3. Synthesis and Separation of **CB[6]**, **CB[7]**, and **CB[8]**

**Figure 3:**
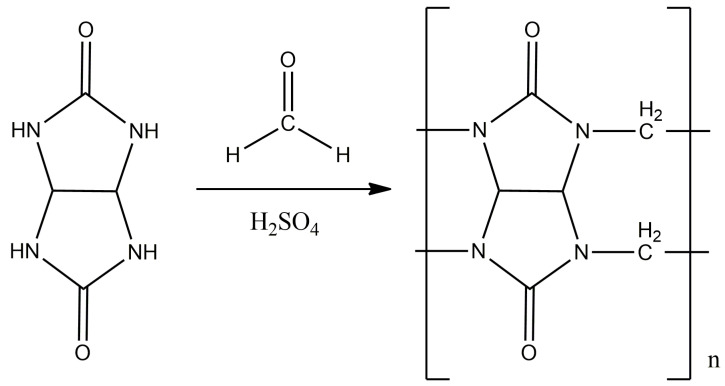
The synthesis scheme of **CB[n]**, where n = 6, 7, 8.

The synthesis was carried out according to the displayed method (Figure 3) [24,25]. A total of 4.22 g (0.14 mol) of paraformaldehyde and 14 mL of 10 M sulfuric acid were placed in a three-neck flask equipped with a magnetic stirrer and a reverse refrigerator, and mixed until the paraform was completely dissolved. Then, 10 g (0.07 mol) of glycoluryl was gradually added to the flask in small portions in order to avoid premature oligomerization. The reaction mass was thermostated and kept for 24 h at a temperature of 95 °C.

The reaction mass was evaporated to dry, and the resulting dry substance was ground to a powder, which was then dissolved in 150–200 mL of water and kept for a day with constant stirring. The solution was filtered to obtain masterbatch solution No. 1, and the precipitate was again placed in 150–200 mL of water and kept for a day at a temperature of 40 °C with constant stirring, after which it was filtered again to obtain masterbatch solution No. 2 and precipitate No. 1.

Masterbatch solutions No. 1 and No. 2 were combined and evaporated to 10–15 mL, obtaining solution No. 3.

Separation of **CB[7]**. A total of 80 mL of methanol was added to solution No. 3 and the mixture was mixed for 24 h, observing precipitation; it was then filtered and placed in a vacuum cabinet for 12 h at a temperature of 60 °C. The resulting powder was studied using NMR and XRD. Next, precipitate No. 1 was dissolved in 15 mL of a 40% formic acid solution, and the undissolved part was filtered out—this is precipitate No. 2, and the masterbatch solution was solution No. 4.

Separation of **CB[8]**. Precipitate No. 2 was recrystallized from concentrated HCl, and crystalline **CB[8]** was obtained. The structure was confirmed by NMR and XRD methods.

Separation of **CB[6]**. Solution No. 4 was evaporated to dry, and the precipitate was washed with 20 mL of water and then recrystallized from 8 M HCl to obtain crystalline **CB[6]**.

**Figure 4:**
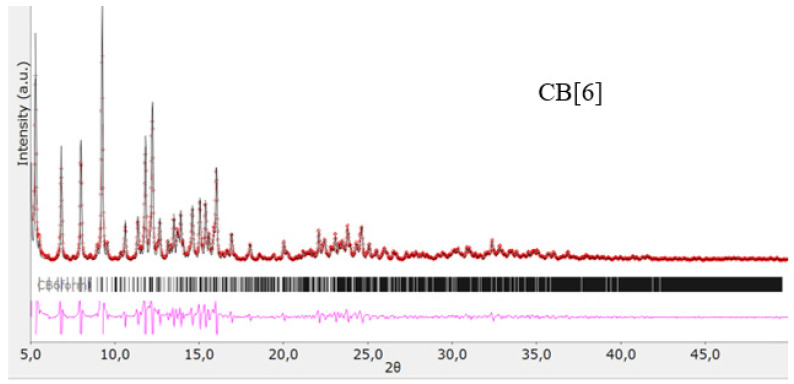
Diffractogram of **CB[6]**, red color—obtained substance, black—standard.

**Figure 5:**
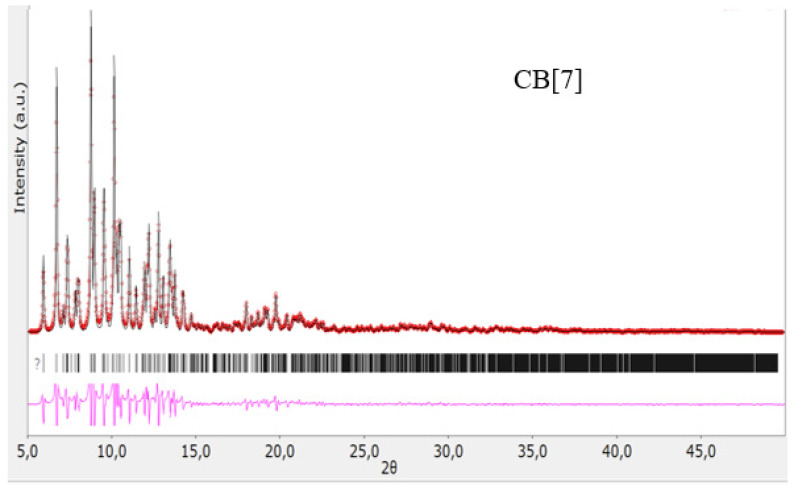
Diffractogram of **CB[7]**, red color—obtained substance, black—standard.

**Figure 6:**
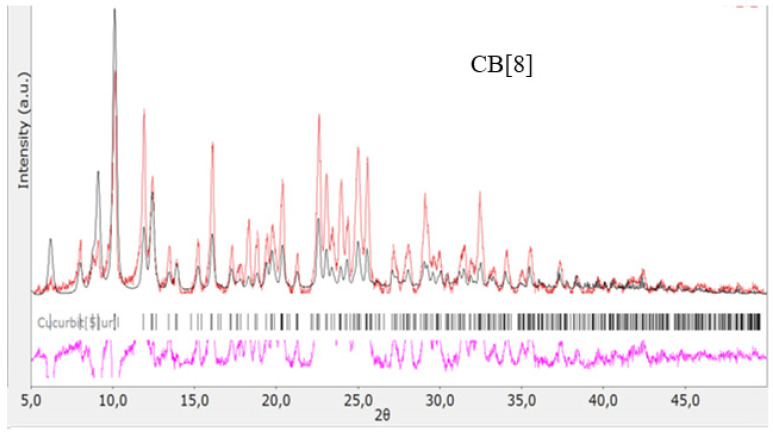
Diffractogram of **CB[8]**, red color—obtained substance, black—standard.

The structures of the obtained cucurbit[n]urils were proved using rentgenophase analysis by comparing the diffractograms obtained by us with the diffractograms available in the Cambridge Crystallographic Data Center database: **CB[6]** No. 7209204, **CB[7]** No. 1428201, and **CB[8]** No. 883371. The crystalline powders of cucurbituriles were kept in a vacuum drying cabinet at a temperature of 70 °C for 24 h to remove crystallization water and hydrochloric acid. Diffractograms of cucurbit[n]urils are shown in Figure 4, Figure 5 and Figure 6. The coincidence of the diffractograms obtained by us when compared with known data not only confirms the structures of the obtained cucurbit[n]urils, but also indicates their sufficient division into individual macrocycles for further work with them.

### 2.4. Applying **CB[n]** to **HA**

**Figure 7:**
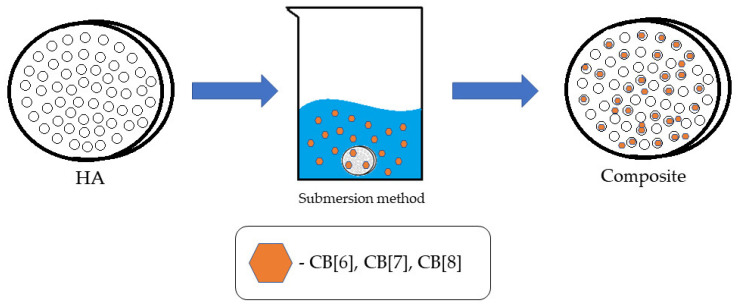
Illustration of the methods of applying **CB[n]** to **HA**.

The method of immersion in solution was used for the application (Figure 7). To implement it, the tablet from **HA** was immersed in solutions in **CB[n]** water for 45 min with a concentration of 1 mg/mL. Solutions of **CB[6]** and **CB[8]** were dispersed when **CB[7]** was completely dissolved in water. In parallel, a blank experiment was conducted—with distilled water instead of a solution with a bioactive substance to obtain comparison samples. The obtained samples were dried at room temperature to a constant weight for 24 h.

The quantitative deposition of **CB[n]** was also investigated. To do this, the matrix solution was drained and evaporated. The resulting precipitate was calcined to a constant mass and weighed. The mass of the applied **CB[n]** ranged from 8 to 10 mg.

### 2.5. Assessment of Hemocompatibility of Biocomposites

One of the ways to assess overall cytotoxicity is to study hemolytic activity [26].

In this study, samples were used, which are pressed scaffolds made of hydroxyapatite (**HA**), shaped like a cylinder with a diameter of 20 mm and a height of 2 mm. The samples were pressed hydroxyapatite tablets with cucurbit[n]urils applied (**CB[6]**, **CB[7]**, and **CB[8]**).

Modification of the surface of the samples submitted for study was the application of **CB[n]** on them, and this was carried out by immersion in solution. In this case, a 1% solution of **CB[n]** in distilled water was used. The exposure time in all cases was 40 min. The samples were immersed in a solution in **CB[n]** followed by air drying at room temperature for 24 h. This method was chosen because of its high efficiency and low labor costs compared to instrumental application methods (using ultrasonic sound, vacuum blowing, or a microwave reactor).

The standard procedure prescribed by ISO 10993-4:2017 was used to assess the hemocompatibility of the samples [27]. To assess the hemocompatibility of the samples, whole hemostated blood from a healthy donor was used. The blood was centrifuged and the erythromass was separated. The resulting erythromass was diluted with a sterile solution of 1×PBS with a temperature of 37 °C in a ratio of 1:9. The samples were placed in a standard 12-well cell culture plate and filled with the resulting blood solution in PBS in a ratio of 1 mL of solution per 1 cm^2^ of the sample surface area. Deionized water was used as a positive control (100% hemolysis), and a solution of 1× PBS (0% hemolysis) was used as a negative control. An unmodified hydroxyapatite was used as a control material. The tablet was then incubated in a thermostat at 37 °C for 60 min. After that, the blood from the wells of the tablet was transferred to centrifuge tubes and centrifuged for 5 min at 3000 rpm to precipitate the remaining red blood cells. Then, the supernatant was carefully removed and transferred to a standard 96-well plate for spectroscopic analysis, and the optical density was read using a Tecan Infinite F50 IFA reader (Grödig, Austria) at 492 nm. The percentage of hemolysis was the average of three repetitions and was calculated using the following formula [27]:

$$\mathrm{Hemolisys},\;\%=\frac{OD_{\mathrm{test}}-OD_{\mathrm{control}}^{\mathrm{negative}}}{OD_{\mathrm{control}}^{\mathrm{positive}}-OD_{\mathrm{control}}^{\mathrm{negative}}}\times 100\%$$

### 2.6. Assessment of Cytotoxicity of Biocomposites

To assess the cytotoxicity of the samples in vitro, cells of the immune system were used, namely monocytes isolated from the peripheral blood of healthy donors who gave informed consent. Blood sampling was performed by phlebotomy of the cubital vein. Monocytes were isolated from human peripheral blood according to the method proposed by J. Kzhyshkowska and colleagues [28]. The sterilization of biocomposite samples was carried out by ultraviolet irradiation in lime chloride vapor. After that, the samples were placed in the wells of a 12-well tablet and filled with 1 mL of a nutrient cell medium with monocytes with a cell concentration of 1 = 106 in 1 mL. Incubation was carried out at a temperature of 37 °C and 7.5% CO_2_ for 144 h.

To study the effect of materials on the viability of mononuclear cells, an Alamar Blue test was used [29]. Assessment of the viability of cells of the immune system was carried out after incubation on the surface of the studied materials. After that, a supernatant was taken from each well, leaving 500 µL of medium with cells in the well. Additionally, 50 µL of Alamar Blue reagent was added to the wells (the volume ratio of Alamar Blue–cell medium was 1/10). Cells with Alamar Blue were incubated for 3 h at a temperature of 37 °C in a dark place. After incubation, a cellular medium with Alamar Blue was introduced into the 96-well plate (3 wells for each sample). The intensity of the fluorescence signal was measured using a Tecan Infinite 200 microrider at a wavelength of 540 nm [29]. Upon entering a living cell, the active component of the rezazurin indicator is converted into resorufin, a substance that has pronounced fluorescence in the red region. Thus, the method allows for a comparative analysis of cell viability and quantification [30].

Cells cultured on a substrate without samples were used as a positive control (CTRL 100%). Samples of unmodified hydroxyapatite (**HA**) exposed to the same effect as the experimental ones were used as a reference. The percentage of surviving cells was calculated using the following formula:

$$\mathrm{Living}\;\mathrm{cells},\;\%=\frac{{\mathrm{IF}}_{test}-IF_{\mathrm{control}}^{\mathrm{negative}}}{IF_{\mathrm{control}}^{\mathrm{positive}}-IF_{\mathrm{control}}^{\mathrm{negative}}}\times 100\%$$

where IF is the fluorescence intensity.

### 2.7. Evaluation of the Proinflammatory Properties of Biocomposites and Their Effect on Monocyte Activation

The effect of the samples on the activation of human peripheral blood monocytes was assessed by enzyme immunoassay (ELISA). Enzyme immunoassay was performed in accordance with the manufacturer’s recommendations (Vector-BEST, Novosibirsk, Russia). To do this, a supernatant was selected from each well, leaving 500 µL of medium with cells in the well to assess viability according to the procedure described above. To study the immunomodulatory properties, the supernatants were stored at minus 80 °C.

### 2.8. Statistical Analysis

Statistical analysis was performed using STATISTICA 8.0 for Windows (STATISTICA, RRID: SCR_014213, Tibco, Palo Alto, CA, USA). The Mann–Whitney test and *t*-test for independent groups were implemented. The data were checked for normality of distribution using the Shapiro–Wilk statistical criterion. Results were considered to be significant with *** *p* < 0.001, ** *p* < 0.01, and * *p* < 0.05.

## 3. Results and Discussion

In this study, we explored the impact of macrocyclic compounds, specifically **CB[n]**, on the biocompatibility of hydroxyapatite (**HA**), with the aim of developing a matrix for promising biocomposite materials suitable for medical applications. The materials obtained were subjected to thorough characterization using infrared (IR) spectroscopy, scanning electron microscopy (SEM), and energy-dispersive X-ray fluorescence spectroscopy (EDS).

**Figure 8:**
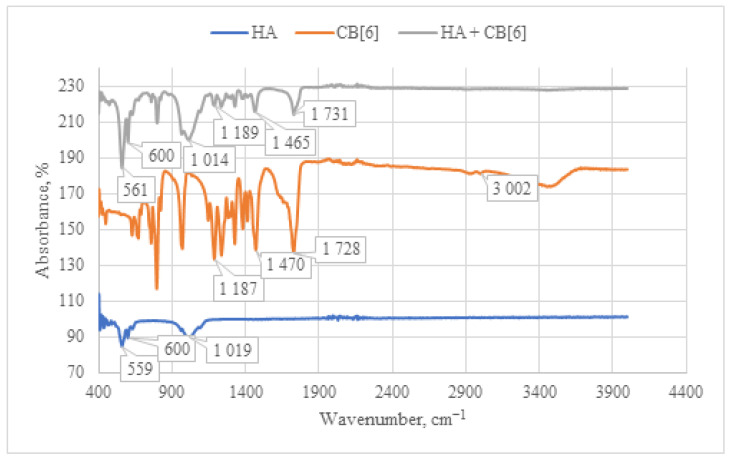
Comparative IR spectrum of the sample **HA + CB[6]** (gray line), **HA** (blue line), and **CB[6]** (orange line).

When analyzing the IR spectrum of the **CB[6] + HA** composite obtained through immersion (as shown in Figure 8), characteristic absorption bands of **CB[6]** are observed. Specifically, peaks at 1731 cm^−1^ are evident, corresponding to the valence vibration of carbonyl groups (C=O) present in the glycoluril linkage of **CB[6]**. In the IR spectrum, deformation vibrations of the CH_2_ groups are detected in the range of 1200–1500 cm^−1^, along with vibrations of the OH groups at 3400–3500 cm^−1^. These OH vibrations correspond to both water molecules and hydroxyl groups of **HA**. The concurrent presence of these characteristic vibrations confirms the incorporation of **CB[6]** into the composition of the **HA + CB[6]** composite.

**Figure 9:**
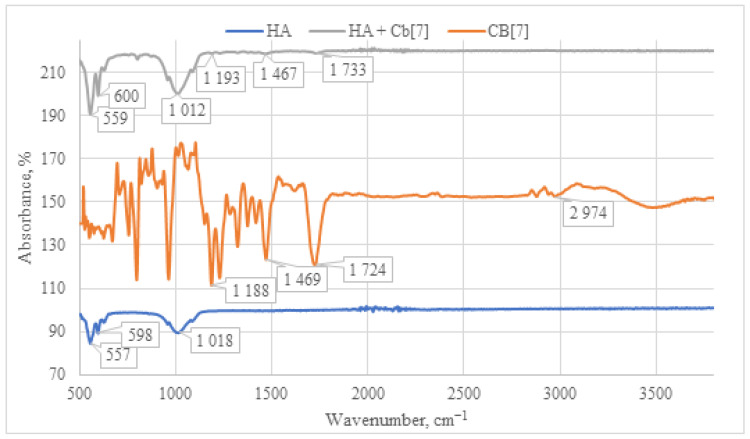
Comparative IR spectrum of the sample **HA + CB[7]** (gray line), **HA** (blue line), and **CB[7]** (orange line).

The IR spectrum of the **HA + CB[7]** sample exhibits a similar pattern to that of **HA + CB[6]** (as depicted in Figure 9), albeit with significantly lower signal intensity. This reduced intensity can be attributed to the high solubility of **CB[7]** in water. Consequently, the lower quantity of **CB[7]** on the surface leads to diminished signal intensity in the IR spectrum. This observation is further supported by SEM and EDS analyses, as depicted in Figures 12 and 13, which confirm the lower presence of **CB[7]** on the surface compared to **CB[6]**. Moreover, a notable shift of the carbonyl group C=O signal is observed in the **HA + CB[7]** sample, amounting to 7 cm^−1^ towards the long-wavelength region. In contrast, the shift observed in **HA + CB[6]** (as depicted in Figure 8) is only 3 cm^−1^. This discrepancy suggests a subtle interaction between **CB[7]** and the **HA** surface.

**Figure 10:**
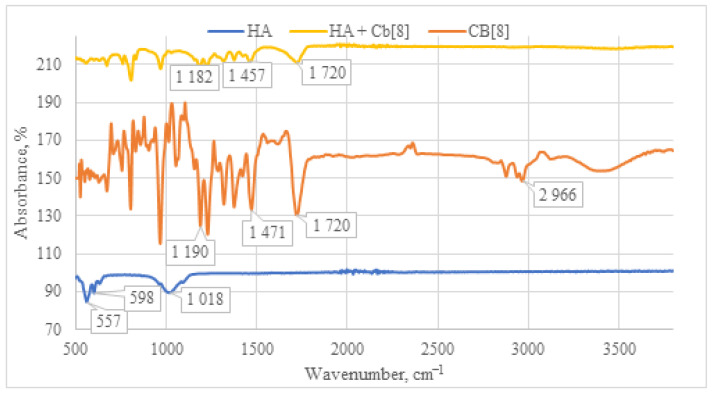
Comparative IR spectrum of the sample **HA + CB[8]** (yellow line), **HA** (blue line), and **CB[8]** (orange line).

Upon examination of the IR spectrum of the **HA + CB[8]** sample (Figure 10), a pattern analogous to **HA + CB[6]** is observed. All characteristic absorption bands for **CB[8]** are present, including the C=O signal at 1720 cm^−1^, along with deformation vibrations of CH_2_ groups within the 1200–1500 cm^−1^ range. Taken together, these observations affirm the presence of **CB[8]** in each of the samples.

**Figure 11:**
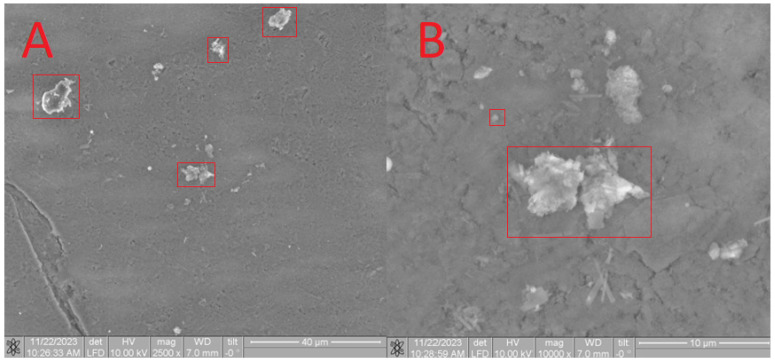
SEM image of the **CB[6] + HA** sample, approximating 40 microns (**A**) and 5 microns (**B**).

Furthermore, SEM analysis was conducted to characterize the obtained composites. On the surface of the **HA** in the **CB[6] + HA** sample, distinct **CB[6]** particles are prominently observed (Figure 11A), ranging in size from 10 microns to 700 nm. These **CB[6]** particles are highlighted within the red square in the image.

Upon closer scrutiny, ensembles of **CB[6]** molecules are distinctly observed (Figure 11B), aligning with the dimensions of the pores. These ensembles exhibit diameters reaching up to 700 nm, as indicated by the red square in the image. The formation of such ensembles can be attributed to the propensity of **CB[6]** to form dispersed solutions in water. The diameter of certain **CB[6]** particles (highlighted by the red square) exceeds the pore size of the hydroxyapatite (**HA**) carrier, leading to overlapping pores where the **CB[6]** macrocycles remain on the surface. This uneven distribution across the surface arises from the partial solubility of **CB[6]**, causing some particles to penetrate into the surface. This penetration is confirmed by infrared spectroscopy of the scaffold chip.

**Figure 12:**
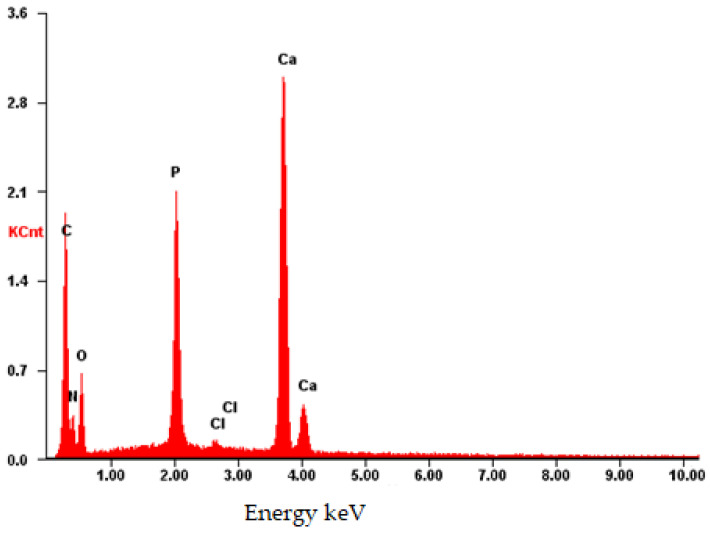
EDS of the **CB[6] + HA** sample.

The composition was further validated using energy-dispersive X-ray fluorescence spectroscopy (EDS). As illustrated in Figure 12, aside from the predominant elements of hydroxyapatite (P and O), the presence of N and C atoms is evident. This observation confirms that the substance on the surface possesses an organic nature and is distinct from the carrier material.

**Figure 13:**
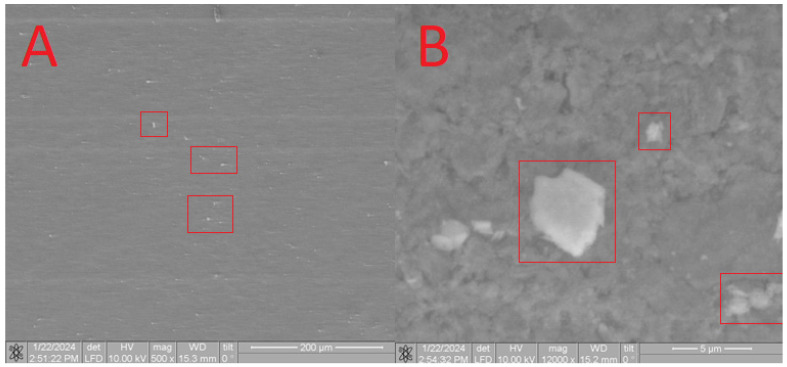
SEM image of the **CB[7] + HA** sample, approximating 200 microns (**A**) and 5 microns (**B**).

Upon analysis of the **CB[7] + HA** sample (Figure 13), a distinct pattern is observed, which correlates with the complete dissolution of **CB[7]** in water. Consequently, during drying, only small particles ranging from 100 to 500 microns were formed on the surface. The presence of **CB[7]** is further evidenced by the observed increase in biocompatibility (as depicted in Figures 17–20). In its dissolved state, **CB[7]** can penetrate more effectively into the material, facilitating slower release from the material’s pores and, therefore, prolonging its action.

**Figure 14:**
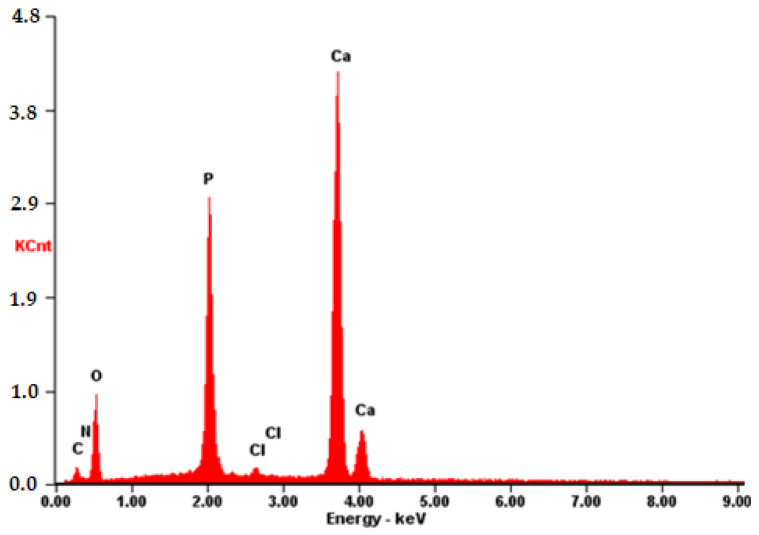
EDS of the **CB[7] + HA** sample.

The EDS spectrum exhibits robust signals corresponding to Ca, P, and O, consistent with the predominant elements of hydroxyapatite. Despite the dominance of these elements, the spectrum also detects the presence of N and C atoms, although their signals may be less prominent due to overlapping with the signals from Ca, P, and O. Nonetheless, the detection of N and C atoms confirms their presence on the surface, as depicted in Figure 14.

**Figure 15:**
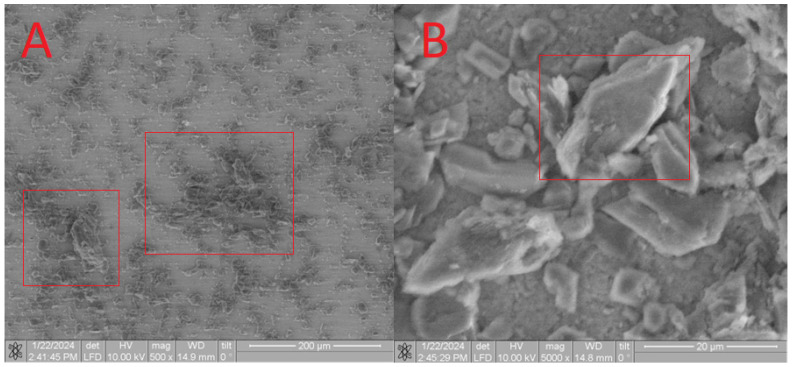
SEM image of the **CB[8] + HA** sample, approximating 200 microns (**A**) and 20 microns (**B**).

Upon examination of the **CB[8] + HA** sample, a distinct observation is made. **CB[8]** disperses easily in an aqueous solution and is applied to the surface more uniformly (as shown in Figure 15), forming larger conglomerates of molecules ranging from 7 to 20 microns in size. This characteristic enables an increase in the effective surface area of the material, thereby enhancing the overall efficiency of the material.

**Figure 16:**
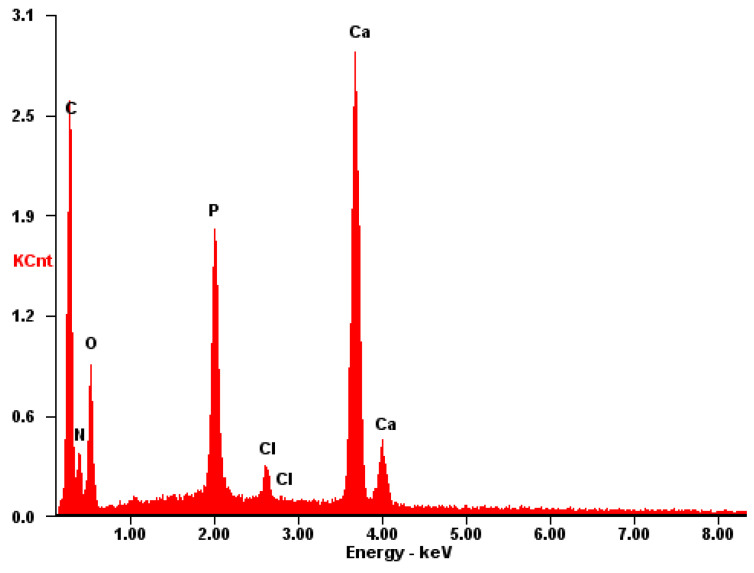
EDS of the **CB[8] + HA** sample.

The EDS spectrum of the **CB[8] + HA** sample (Figure 16) exhibits prominent signals associated with the surface composition of HA, including Ca, P, and O. Furthermore, the presence of large conglomerates of **CB[8]** molecules is evident from clear signals of N and C atoms. These findings corroborate the earlier results obtained from Figure 8, Figure 9 and Figure 10, confirming the successful incorporation of **CB[8]** into the composite material.

In the subsequent phase of our investigation, we delved into the biological properties of the synthesized composites. The data presented in Table 2 indicate that all the tested samples of biocomposite materials exhibited hemolytic activity, a finding supported by statistical analysis (*p* < 0.01). Notably, both the modified **CB[n]** samples and their unmodified counterparts displayed hemolytic activity. The investigation revealed that the hemolytic activity of the **HA + CB[6]**, **HA + CB[7]**, and **HA + CB[8]** samples falls below 5%, aligning with the requirements for medical materials [27]. Furthermore, statistical analysis indicated that the hemolytic activity of these samples did not significantly differ from that of unmodified hydroxyapatite (**HA**) (*p* > 0.05). Notably, the **HA + CB[8]** (HCl) samples displayed the highest level of hemolytic activity among the groups tested in Table 2, likely due to their lack of specific purification from HCl. This discrepancy can be attributed to the presence of hydrochloric acid, an impurity in the synthesis medium for the entire **CB[n]** series, when the feedstock is inadequately purified. Hydrochloric acid can significantly increase the hemolytic effect and pose considerable harm to the body. Therefore, in material preparation, it is crucial to minimize harmful impurities. A comparison between the purified sample (**HA + CB[8]**) and the untreated sample reveals a notable difference in hemolytic effect, aligning with the broader trends observed across the **CB[n]** series.

As per literature data, plasma proteins exhibit a propensity to adsorb onto any abiotic surface, with the composition of the adsorbed protein layer being notably influenced by surface charge and potential disparities. Specifically, if there is a positive potential difference between the medical material and the blood, the risk of thrombosis may increase [6]. It is hypothesized that the treatment methods employed for hydroxyapatite in this study may lead to a reduction in the positive potential difference between the sample surface and blood, consequently resulting in a decrease in hemolysis. Conversely, immersion-based treatment may not significantly mitigate the positive potential difference, potentially contributing to hemolysis.

The hemolytic activity of unmodified hydroxyapatite (**HA**) in relation to cucurbit[n]urils may also be influenced by its porosity. Functionalization of hydroxyapatite with various substances can lead to a reduction in porosity. According to the contemporary literature, the manifestation of hemolysis on inert material surfaces is intricately associated with the adsorption of plasma proteins, particularly fibrinogen, onto the material’s surface in direct interaction with blood. Heightened levels of plasma protein adsorption are commonly correlated with increased hemolytic activity [31,32]. Hydroxyapatite is characterized by its porous nature with relatively small pore sizes, typically smaller than the size of protein molecules. Consequently, the adsorption of plasma proteins onto hydroxyapatite is considered to be insignificant [31]. It is reasonable to assume that this factor contributes to the low level of hemolysis observed in samples of unmodified hydroxyapatite (**HA**).

According to the literature findings, **CB[n]** without a carrier exhibits low toxicity towards various cell types [33]. Research has shown that **CB[n]** does not induce hemolysis, even at high concentrations (1 mM), under PBS conditions. Nevertheless, investigations have unveiled that **CB[n]** exhibits the capacity to elevate the level of early apoptosis, which is associated with the presence of phosphatidylserine on the cell surface. Remarkably, even under these conditions, the integrity of the cell membrane remained unaffected. Additionally, **CB[7]** has been observed to induce hemolysis in the presence of albumin, albeit not under PBS conditions, except for **CB[7]** at a concentration of 2 mM. It is worth noting that **CB[7]**, along with other homologs (excluding **CB[5]** and **CB[6]**), have the capability to bind cholesterol molecules. Thus, the hemolytic impact induced by cucurbiturils may mirror the effects observed with cyclodextrins [34].

Hemolysis in the presence of albumin medium is primarily attributed to the indirect interaction of **CB[n]** with the components of the medium rather than the direct toxic effect of cucurbiturils themselves. It is established that **CB[7]** has the capability to form complexes with amino acids, peptides, and proteins [35]. However, due to its lower solubility, **CB[8]** was employed in lower concentrations compared to **CB[7]**. Hence, **CB[7]** at the employed concentration may interact with amino acid residues within albumin. Presumably, this interaction facilitates the delivery of **CB[7]** to red blood cells, resulting in cellular demise. The heightened hemolytic activity observed in the presence of **CB[7]** necessitates further exploration. Notably, this effect was not apparent in serum containing equivalent albumin concentrations, suggesting that a similar outcome may only manifest in experimental in vitro setups.

It is noteworthy that previous research [36] utilized **CB[n]** concentrations ranging from 2 to 0.01 mM, revealing **CB[n]** to be well-tolerated by cells at concentrations up to 0.3 mM. Concentrations exceeding 0.3 mM may not be required for drug delivery systems. The absence of such an effect in **CB[6]** is likely attributable to its smaller cavity size and distinctive chemical and biological properties, enabling effective binding to biologically active molecules within the experimental milieu. Therefore, the cucurbiturils examined in our study do not directly harm cells but have the potential to influence components of the cellular microenvironment [36].

It is imperative to emphasize the persistent challenge of undesired blood clot formation upon contact with implantable materials and devices, which remains unresolved. This issue arises due to the presence of protective mechanisms within a healthy vascular endothelium that resist thrombosis, mechanisms which are lacking in foreign materials introduced into the body. Instead, biomaterials often promote blood coagulation through the activation of a series of interconnected processes, including protein adsorption, platelet and leukocyte adhesion, thrombin production, and complement activation [36]. Therefore, the quest for methods that do not elevate the percentage of hemolysis in the functionalization of biocompatible materials is particularly pertinent.

### 3.1. Assessment of Cytotoxicity of Biocomposites

**Figure 17:**
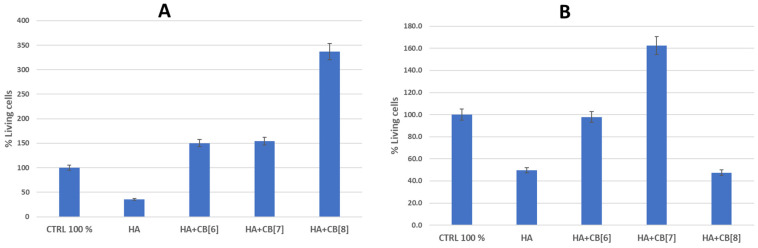
The effect of biocomposites on the viability of cells of the immune system. (**A**) is donor 1; (**B**) is donor 2. **HA**—hydroxyapatite; **CB[6]**—cucurbite[6]uril; **CB[7]**—cucurbite[7]uril; **CB[8]**—cucurbite[8]uril.

Based on the results of the hemocompatibility study, the cytotoxicity of several biocomposite materials (**HA + CB[6]**; **HA + CB[7]**; and **HA + CB[8]**) for immune system cells was investigated (Figure 17).

As follows from the data in Figure 16, the sample of unmodified hydroxyapatite (**HA**) had a toxic effect on the cells of the immune system of both donors. Perhaps this is a consequence of the insignificant background cytotoxicity of hydroxyapatite, which was confirmed as a result of experiments on the study of hemocompatibility of samples [31,37]. This observation could be attributed to the high surface energy of **HA**. Although pure **HA** exhibits notable cytotoxic properties, the inclusion of cucurbit[n]urils can notably decrease surface energy, enhancing cell viability, and consequently improving biocompatibility.

The **HA + CB[6]** sample, on the contrary, augmented the survival of immune system cells by 50.4% in donor 1 and showed a marginal decrease of 2.2% in donor 2 compared to the positive control. **HA + CB[7]** enhanced the survival rate of immune system cells by 54.0% in donor 1 and by 62.3% in donor 2. Meanwhile, **HA + CB[8]** elevated the number of cells surviving after incubation by 236.8% in donor 1, yet conversely decreased the number of cells by 52.5% in donor 2 compared to the positive control.

Based on the data obtained, all the examined samples of biocompositional materials (**HA + CB[6]**, **HA + CB[7]**, and **HA + CB[8]**) demonstrated high biocompatibility with both erythrocytes and mononuclear cells.

It is evident that the functionalization of hydroxyapatite with macrocyclic nitrogen-containing substances of the **CB[n]** family decreases its sorption capacity and the affinity of plasma proteins to the material’s surface. Consequently, this leads to an enhancement in biocompatibility [31]. Discrepancies in the survival rates of cells obtained from various donors after incubation with the tested materials could stem from differing levels of immune reactivity.

### 3.2. Evaluation of the Proinflammatory Properties of Biocomposites and Their Effect on Monocyte Activation

The selection of key macrophage cytokines TNF-α, IL-1β, IL-6, and IL-10 as the main parameters for studying immunomodulatory properties aligns with the model system employed in the study. Since the biological activity of cytokines primarily depends on their concentration in the extracellular medium rather than the level of gene expression, enzyme immunoassay was employed as the primary method to determine the effect of scaffolds on cytokine production.

**Figure 18:**
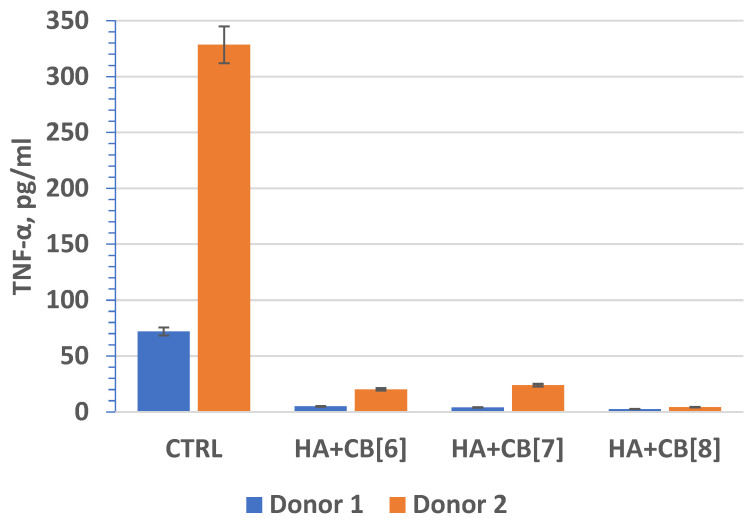
The effect of biocomposite samples on TNF-α production by primary human monocytic macrophages.

Tumor necrosis factor (TNF, TNF-α) (Figure 18) is an extracellular protein, serving as a multifunctional proinflammatory cytokine primarily synthesized by monocytes and macrophages. TNF plays a crucial role as the main mediator of inflammation and serves as an important regulator of the immune response in reaction to infection and tumor development. Beyond its inflammatory functions, TNF-α also impacts lipid metabolism, coagulation, insulin resistance, and endothelial function. Furthermore, TNF-α stimulates the production of other cytokines, such as IL-1, IL-6, IL-8, and interferon-gamma, and activates leukocytes, contributing significantly to defense against intracellular parasites and viruses.

**Figure 19:**
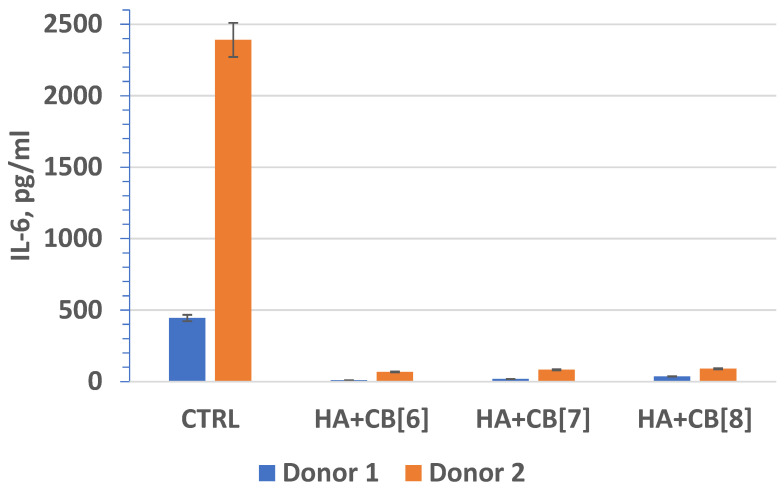
The effect of biocomposite samples on the production of IL-1β by primary human monocytic macrophages.

Interleukin 1 beta (IL-1β) (Figure 19) is a cytokine known for regulating the progression of inflammatory reactions. Its biological role encompasses various effects, including immunomodulatory, hematopoietic, inflammatory, and intersystem functions. IL-1β plays a critical role in initiating the early stages of the immune response, involving specific T-lymphocytes, particularly T-helper cells, in the process. Indeed, interleukin 1 beta (IL-1β) plays a pivotal role in various immune processes. It promotes the differentiation of B lymphocytes into plasma cells, expediting antibody production. IL-1β is among the earliest responders in the body’s defense against pathogens, activating and regulating inflammatory and immune responses. It activates neutrophils and T and B lymphocytes, and also enhances the synthesis of acute phase proteins. Additionally, IL-1β boosts phagocytosis, hematopoiesis, and vascular permeability, and exhibits cytotoxic and bactericidal activities. The inflammatory function of IL-1β is absolutely significant. It amplifies the motility of neutrophils, augments cellular activity within inflammatory sites, and enhances the activity of other cytokines [38,39], contributing to the overall inflammatory response.

**Figure 20:**
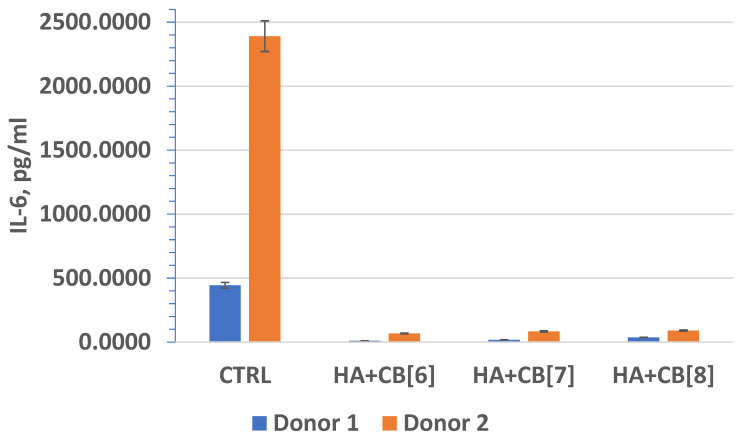
The effect of biocomposite samples on the production of IL-6 by primary human monocytic macrophages.

In fact, interleukin-6 (IL-6) (Figure 20) plays a crucial role as a multifunctional cytokine. It participates in the differentiation of activated B lymphocytes into plasma cells that secrete immunoglobulins, and it regulates the acute phase response. Elevated levels of IL-6 have been observed in numerous inflammatory conditions, often correlating with laboratory markers of inflammatory activity [40].

Interleukin-10 (IL-10) is a vital cytokine with unique properties. It belongs to Class 2 cytokines and exhibits powerful anti-inflammatory effects by suppressing the production of proinflammatory cytokines, such as IFNy, tumor necrosis factor α (TNFa), IL-1β, and IL-6, in various cell types. Additionally, IL-10 hinders the maturation of dendritic cells, partly by suppressing IL-12 expression [41]. Absolutely, interleukin-10 (IL-10) demonstrates a multifaceted role in immune regulation. In addition to its anti-inflammatory properties, IL-10 also exhibits immunostimulatory effects. It can stimulate the production of IFNy in CD8+ T cells that are activated by anti-CD3/anti-CD28 or other cytokine cocktails. Moreover, IL-10 serves as a potent growth and differentiation factor for various immune cells, including B cells, mast cells, and thymocytes [42].

In fact, these findings are significant as they suggest that the biocomposites (**HA + CB[6]**, **HA + CB[7]**, and **HA + CB[8]**) have the potential to be used in biomedical applications without inducing or exacerbating inflammatory responses. This is essential for ensuring the safety and efficacy of such materials in various medical contexts, ranging from tissue engineering to drug delivery systems. The lack of stimulation of proinflammatory cytokine expression indicates the promising biocompatibility properties of these materials, which is crucial for their successful translation into clinical use. The finding that the concentration of IL-10 exceeded the lower limit of sensitivity of the analysis suggests that the materials studied may have induced an anti-inflammatory response. This is promising, as it indicates that the biocomposites (**HA + CB[6]**, **HA + CB[7]**, and **HA + CB[8]**) could potentially modulate the immune response towards an anti-inflammatory phenotype.

The absence of significant differences in the level of cytokine expression among the different materials suggests that they do not induce inflammation or provoke an immune response, at least under the experimental conditions tested. However, individual differences in immune reactivity may influence cytokine expression levels, highlighting the importance of considering patient-specific factors in the evaluation of biocompatibility and immunomodulatory effects of biomaterials.

## 4. Conclusions

The study’s findings highlight the potential of using conglomerate ensembles of **CB[n]** molecules to enhance the biocompatibility of materials, particularly when deposited on porous substrates, like **HA**. **HA**, known for its biocompatibility and porosity, serves as an excellent matrix for such modifications. By depositing nitrogen-containing macrocyclic cavitands, such as **CB[6]**, **CB[7]**, and **CB[8]**, onto the surface of porous materials, like **HA**, it becomes feasible to create promising biomaterials conducive to osteogenesis while enabling controlled release of biologically active substances, including antibacterial drugs and other therapeutic agents.

The study has successfully developed a novel method for functionalizing the surface of hydroxyapatite using macromolecular nitrogen-containing heterocycles, specifically **CB[6]**, **CB[7]**, and **CB[8]**. In vitro assessments have demonstrated the high biocompatibility and low toxicity of the resulting biocomposite materials. These innovative biocomposites hold promising potential for finely modulating biological responses, facilitated by the incorporation of **HA + CB[6]**, **HA + CB[7]**, and **HA + CB[8]** on specialized media. Further research will delve into exploring and harnessing the full capabilities of these materials in various biomedical applications.

The comprehensive studies conducted on materials based on macrocyclic compounds, including **CB[n]** and **Bu[6]**, have revealed a plethora of potential applications for these materials. Their unique ability to serve as containers for medicinal or biologically active substances presents an urgent and promising avenue for further exploration. Leveraging the properties of **CB[n]** and **Bu[6]** to encapsulate therapeutic agents opens up exciting possibilities in drug delivery systems and other biomedical applications. This research paves the way for advancements that could significantly impact various fields, including medicine and biotechnology.

## Figures and Tables

**Table 1:** Phase composition of **HA** synthesis products.

Sample	The Inorganic Phase	Parameters of the Electronic Cell, Ǻ	
		a	c
Synthesis product (**HA**)	Ca_10_(PO_4_)_6_(OH)_2_	9411	6863
JCPDS data, No. 9-432	Ca_10_(PO_4_)_6_(OH)_2_	9418	6884

**Table 2:** The level of hemocompatibility of biocomposite samples.

No.	Sample	% of Hemolysis
1	**HA + CB[6]**	1.8665 ± 0.0008 *
2	**HA + CB[7]**	2.0138 ± 0.0019 *
3	**HA + CB[8]**	1.7611 ± 0.0050 *
4	**HA**	1.6004 ± 0.0018 *
5	**HA + CB[8] (HCl)**	44.1611 ± 0.0050 **
6	CTRL (Blood + PBS)	00
7	CTRL (Blood + H_2_O)	100

Note: **HA**—hydroxyapatite; **CB[6]**—cucurbite[6]uril; **CB[7]**—cucurbite[7]uril; **CB[8]**—cucurbite[8]uril. *—the level of hemolysis is statistically significantly different from the negative control (*p* < 0.01). **—the level of hemolysis is statistically significantly different from the negative control (*p* < 0.001).

## Data Availability

The data presented in this study are available in this article.
